# High-resolution laboratory lysimeter for automated sampling of tracers through a 0.5 m soil block

**DOI:** 10.1155/S1463924603000075

**Published:** 2003

**Authors:** A. Johnson, T. J. Mathews, G. P. Matthews, D. Patel, P. J. Worsfold, K. N. Andrew

**Affiliations:** 1 Department of Environmental Sciences Plymouth Environmental Research Centre University of Plymouth Plymouth UK; 2 The National Grid Company Plc Surrey Leatherhead UK

## Abstract

A computer-controlled, automated sample collection from a 0.5-m lysimeter, designed to give superior temporal and spatial resolution for monitoring the movement of chemical tracers through a large undisturbed soil block, is described. The soil block, 0.520.520.5 m, was monitored for saturation using eight time domain reflectometry probes. Rainfall was applied at approximately 1600 ml hm1 using a 12212 array of 23-gauge (0.318 mm internal diameter) hypodermic needles. Soil leachates were collected at the base of the soil block using a machined aluminium collection plate with a 10210 grid of funnels that passed leachates to sample collection palettes. Sample collection was automated using a personal computer equipped with National Instruments LabVIEW™ software and linked to sensors for palette tracking. The automation of the lysimeter allowed sample collection and storage over a user-defined period with no human interaction. As an example of the use of the automated lysimeter, results show the distribution of phosphate within the soil. The eluted phosphate showed an initial and secondary peak, and only emerged from preferential flow channels.


Journal of Automated Methods & Management in Chemistry
Vol. 25, No. 2 (March–April 2003) pp. 43–49

### High-resolution laboratory lysimeter for automated sampling of tracers through a 0.5 m soil block

**A. Johnson[1], T. J. Mathews[1], G. P. Matthews[1]*, D. Patel[2], P. J. Worsfold[1] and K. N. Andrew[1]**

[1] *Department of Environmental Sciences, Plymouth Environmental Research Centre, University of Plymouth, Plymouth, UK*
[2] *The National Grid Company Plc, Leatherhead, Surrey, UK*

*A computer-controlled, automated sample collection from a 0.5-m lysimeter, designed to give superior temporal and spatial resolution for monitoring the movement of chemical tracers through a large undisturbed soil block, is described. The soil block, $0.5 \times 0.5 \times 0.5$ m, was monitored for saturation using eight time domain reflectometry probes. Rainfall was applied at approximately 1600 ml h$^{-1}$ using a $12 \times 12$ array of 23-gauge (0.318 mm internal diameter) hypodermic needles. Soil leachates were collected at the base of the soil block using a machined aluminium collection plate with a $10 \times 10$ grid of funnels that passed leachates to sample collection palettes. Sample collection was automated using a personal computer equipped with National Instruments LabVIEW™ software and linked to sensors for palette tracking. The automation of the lysimeter allowed sample collection and storage over a user-defined period with no human interaction. As an example of the use of the automated lysimeter, results show the distribution of phosphate within the soil. The eluted phosphate showed an initial and secondary peak, and only emerged from preferential flow channels.*

### Introduction

As land uses become more intensive and strictly monitored, there is an increasing need for an improved understanding of pollutant behaviour. There are many approaches used in soil pollutant research, each attempting to gain an increased understanding of how organic- and aqueous-borne pollutants move through a soil structure. The role of preferential flow in soil transport is an area of current investigation by many authors, through the use of either visual methods such as dyes [1] or chemical tracers such as bromide [2]. Preferential flow can be classified as any form of flow that leads to the accelerated dispersal of water and solute through a soil structure, the major source being macropore flow. Macropores (encompassing both cracks and biopores) induce high hydraulic conductivity areas in soil structures [3] and as a consequence, pollutant plumes bypass the bulk of the soil matrix. The relationship between preferential flow zones and the soil–water matrix gives each soil a different reservoir capacity and therefore different consequences with respect to associated land uses.

* To whom correspondence should be addressed. e-mail: P.Matthews@plymouth.ac.uk

The purpose of the apparatus described here is to provide a superior temporal and spatial analysis of leachates from large intact soil blocks, under controlled laboratory conditions, with the objective of generating sufficient experimental data to validate mechanistic models developed to predict pollutant movement through soils. With regard to sample collection, others have used apparatus such as ceramic plates [4] and plastic or metal grids like open trays [5, 6], but none have reported the use of automated sample collection. As discussed below, the rainfall applicator was as good, in terms of evenness of delivery over the surface, as the best applicators described in the literature.

An intact $0.5 \times 0.5 \times 0.5$ m (0.13 m$^3$) cube of undisturbed soil was the largest size sample that could be mounted and studied in a laboratory environment.

The 0.5-m size sample has the advantages for experimentation of being substantially larger than most laboratory cores; hence, more representative of the field scale and providing more stable experimental conditions than field-based trials. Experiments using smaller cores (e.g. $10 \times 50$ cm) can reveal localized phenomena, not necessarily indicative of the soil series as a whole, whereas upscaling of lab-size experiments to field-size modelling demands arbitrary assumptions that can mask the effects of soil structure. In addition, as the length scale is increased, new sources of heterogeneity are encountered, which can cause larger errors. Field scale studies usually have inherently unstable experimental conditions, e.g. fluctuations in properties such as water table movement, bioturbation and transpiration rates. Hence, the objective of the instrumentation reported here is to complement existing field studies, such as those by [7], with more representative laboratory studies at the 0.5 m scale.

### Design and measurement techniques

A 0.5 m block of soil was isolated and extracted for use in the laboratory with the automated lysimeter. After removal of topsoil, the sample was extracted undisturbed by excavating a trench approximately 2 m wide around a column, slightly larger than $0.5 \times 0.5$ m, using a mechanical digger. The sample container was slid over the column in the middle of the pit and excess soil removed as the container was moved down the column. The polycarbonate container had its joints previously sealed with an epoxy gel. A metal base plate was then inserted beneath the container at the depth that the soil was to be extracted. The soil block was then lifted as shown in figure 1 and transported to the laboratory and mounted

*Journal of Automated Methods & Management in Chemistry* ISSN 1463–9246 print/ISSN 1464–5068 online © 2003 Taylor & Francis Ltd
http://www.tandf.co.uk/journals
DOI: 10.1080/1463924031000096507

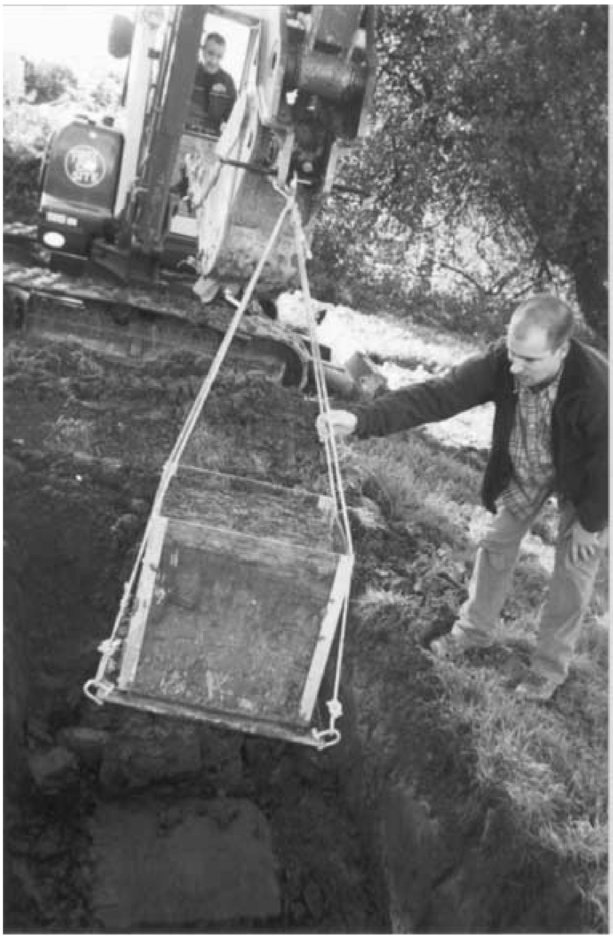

*Figure 1. Extraction of an isolated soil block.*

on the automated lysimeter. The join between the polycarbonate box and aluminium collection plate was sealed with more epoxy resin. Results presented in this paper are from DeBathe soil. The soil is a member of the Crediton series of soils (North Wyke, UK) and is a sandy loam with a high stone content.

The automated lysimeter shown in figure 2 was constructed of square-section steel tubing, with the soil block in the centre of the rig and the rainfall applicator directly above it. Figure 3 shows the top view of the collection plate. The lysimeter sample collection plate was precision machined from anodized aluminium by computer numerate control (CNC). Square funnels with an edge of 38 mm length were machined into the block in a $10 \times 10$ array. Well-defined boundaries reduced the possibility of sampling ambiguity between collection funnels. Each square funnel was filled with glass wool to prevent movement of the soil into the funnels. To prevent samples being biased by edge effects, the machined lysimeter plate had four drainage channels running along each side of the plate. They were 63 mm in width and isolated the central zone of the soil block from which experimental samples were taken. Any water entering the edge channel was removed to waste. The drainage channels left an active surface $380 \times 380$ mm, from which the soil eluates could be collected. The interfaces between the polycarbonate sides of the soil box and the top surface of the soil were sealed with more epoxy resin to prevent excess water flowing down the edge of the sample.

### *Time domain reflectometry (TDR) probes*

The sample was instrumented with TDR probes for measuring soil water content. Two rows of three-pronged probes [8] were inserted horizontally into a soil block at four different depths, the horizontal orientation preventing artificial pathways of flow along the probes themselves [7]. They were constructed of stainless steel welding rods (Rightons, Plymouth, UK), of length 100 mm in column one and 300 mm in column two, and a diameter of 3 mm. The probes were placed with the central point 160 mm from the sample edge at depths of 100, 190, 250 and 400 mm from the soil surface (figure 4). The lowest depth probes were placed so as to avoid any excessive gravity drainage from the base by air intrusion. The outer prongs of the probes were soldered directly to the sheathing of a 1 m coaxial cable, with the central prong being soldered to the copper core of the cable. Each attached section was then wrapped with insulation material.

The other ends of the cables were attached via a BNC connection to a Tektronix 1502B cable tester. Probe measurements were taken at each palette change, which was normally every 4 h.

### Tracer application and rainfall distribution

A method of applying water to the surface of the soil block was required that gave an even distribution over the sample surface. Studies conducted by other authors using spray nozzles found that the uniformity of application deteriorated with horizontal distance from the nozzle [9]. Therefore, it is now more common in the laboratory to use an array of needles. Figure 5 shows this experimental configuration for our rainfall applicator. It produced a variable rainfall application rate over the collection plate with a relative standard deviation (RSD) of 8.8% over 7–8 h, with measurements taken hourly. This compares favourably with other researchers [6, 10–12] (table 1). Rainfall was applied at approximately 1600 ml $h^{-1}$ using a $12 \times 12$ array of 23G (0.318 mm i.d.) hypodermic needles. A degree of horizontal ($x$, $y$) translation was required for the even distribution of rainfall. This was provided by an electric motor attached to a vertical brass rod, upon which a cam was mounted. The cam turned within a PVC ring attached to an edge of the rainfall reservoir, which was supported on roller-ball bearings running on horizontal metal plates. This arrangement was connected to a similar cam on the other side of the apparatus via a chain drive [13].

A constant water pressure head of 34 mm $H_2O$ was supplied to the hypodermic needles via a reservoir made of PVC. An adjustable constant-head device

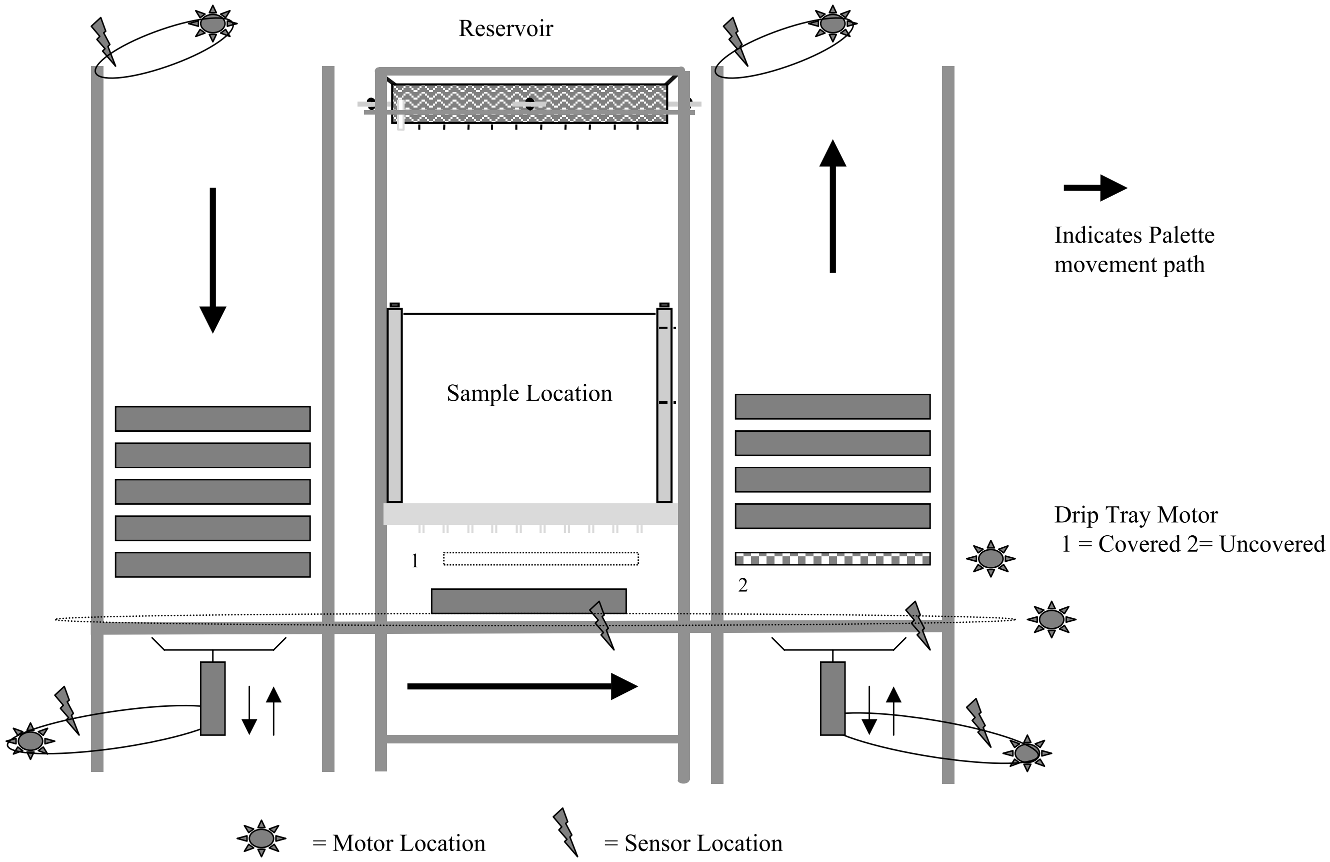

*Figure 2. Schematic diagram of an automated lysimeter with a palette movement path.*

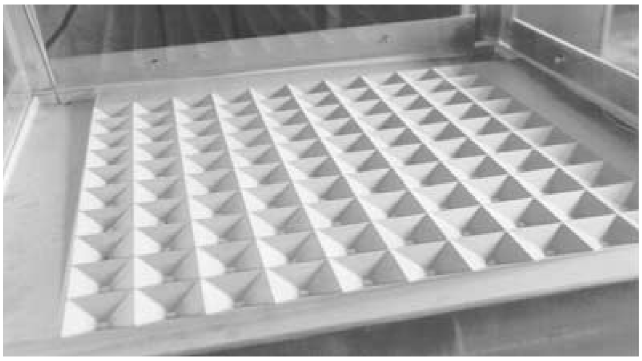

*Figure 3. Precision machined sample collection plate.*

drained away excess water to ensure that the pressure head did not change during the experiment.

Before tracer application, the block was flushed with tap water for 4 days to remove any mobile phosphate remaining in the soil pore water. The tap water was of good quality with a conductivity about 110 μS, and a phosphate level below the detection limit of the air-segmented analyser. Previous experiments using ultra-pure water as a rainfall substitute (Milli-Q$^{TM}$, Millipore Corporation, Watford, UK, 0.0 μS), did not produce a satisfactory phosphate baseline. This was due to the Milli-Q$^{TM}$ water stripping particle-sorbed phosphate from the soil **surface.**

In some lysimeter experiments, sand or soil samples are presaturated from the bottom up, to remove air bubbles and wet all pathways. However, in the present case it was felt that holding the soil at 100% saturation for any time would alter its structure. Therefore, the only preconditioning of the soil was to run the rainfall simulator until a steady flow of water emerged from the bottom of the sample, indicating that saturation equilibrium had been obtained. Water was applied to the sample constantly during the experimental period (typically 48 h), with regular monitoring of the saturation profile using the TDR probes. In later experiments, tracers were applied to the centre of the top surface of the sample in a concentrated slug (1 g in 30 ml). Tracers were bromide (as KBr) and phosphate (as $KH_2PO_4$, BDH, VWR International Ltd, Poole, UK), acting as conservative and non-conservative tracers, respectively.

Leachates from the soil block were analysed using a multichannel air-segmented flow analyser (Sans Plus System®, Skalar, Breda, The Netherlands). This automated instrument had the advantage of a high sample throughput (45 $h^{-1}$), simultaneous bromide and phosphate determination, quality control checks every 10th sample, and on-line calibration during analysis.

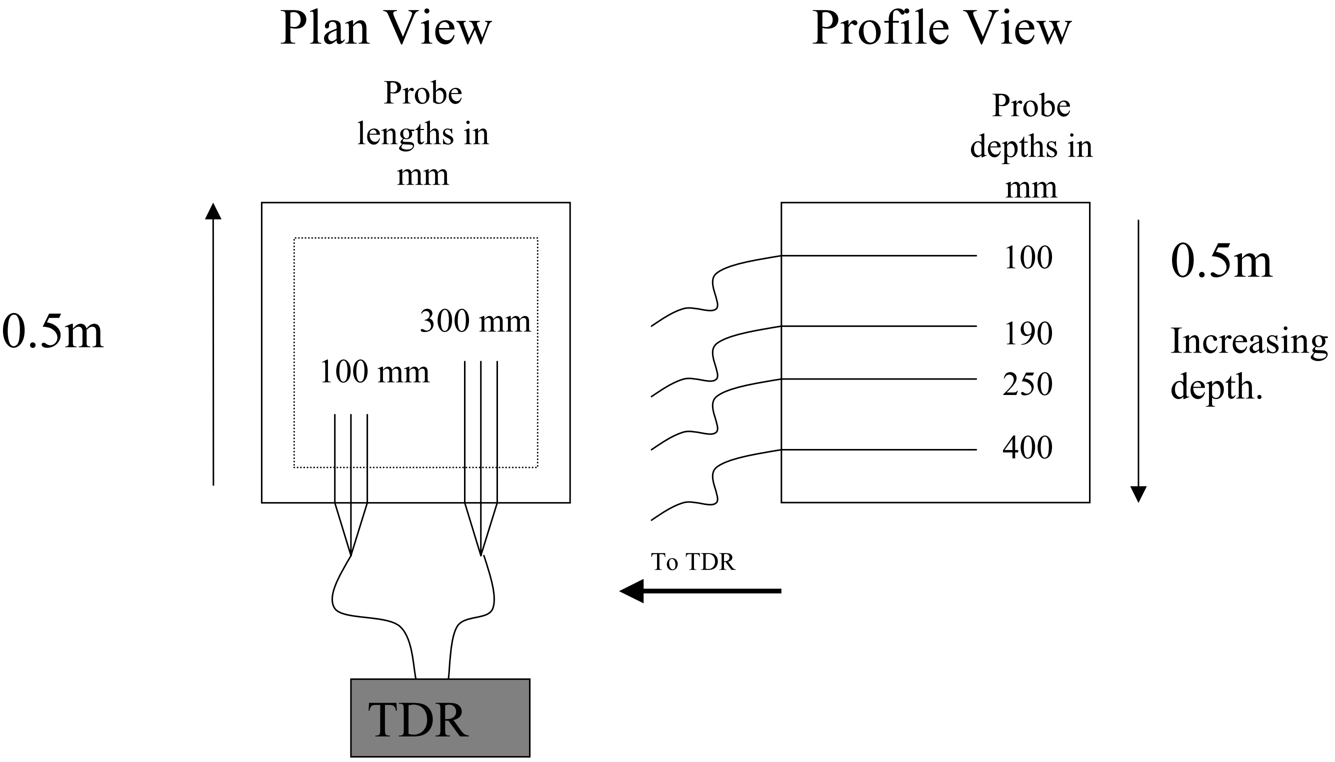

*Figure 4. Time domain reflectometry (TDR) probe distribution in the soil sample.*

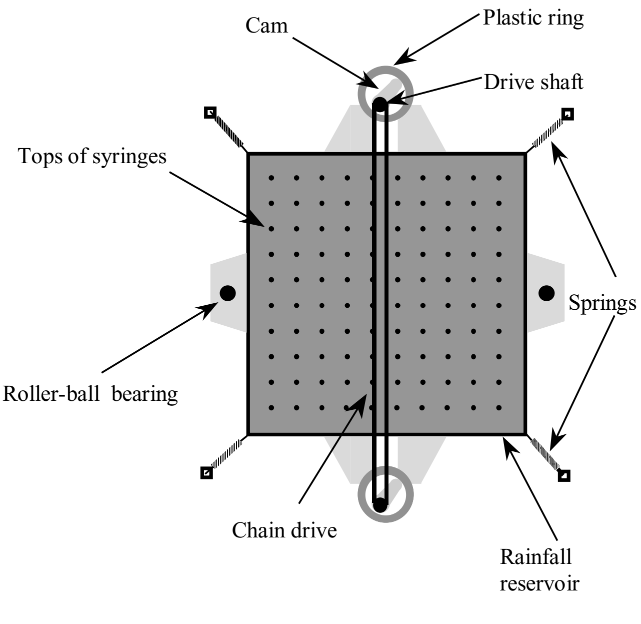

*Figure 5. Schematic of the rainfall distribution rig.*

*Table 1. SD% of rainfall distribution over the collection plate.*

| Workers | RSD % |
|---|---|
| Mathews (1999) [13] | 8.8 |
| Bowman *et al.* (1994) [10] | n/a |
| Dexter (1995) [6] | ≈ 19.0 |
| Phillips *et al.* (1995) [11] | 11.6–22.4 |
| Romkens *et al.* (1995) [12] | 8.5 |

### Sampling system

Funnels protruded from the base of the machined grid lysimeter plate. To prevent cross-contamination of samples, a drip tray was automatically inserted under the funnels during palette changeover and then removed when the sample palette was in position. Palette movement was achieved by a series of chain belts into which the sample trays were mounted. Figure 6 shows the corner arrangement of the sample trays, with the machined gripping areas. Each sample palette comprised a stout PVC tray with the gripping areas machined out of plate steel and attached to the corners of the palette. The upper surface of each palette was drilled with a 10 × 10 grid of holes to hold sample collection vials. The cylindrical glass vials were 30 ml in volume and 25 mm in diameter. The centre of each vial was located below a funnel through which soil eluate could flow.

Sample collection used six electric motors (M1–6) of varying type (220 V DC and 24 V AC, Parvalux, Brighton, UK). Figure 2 shows the motor locations.

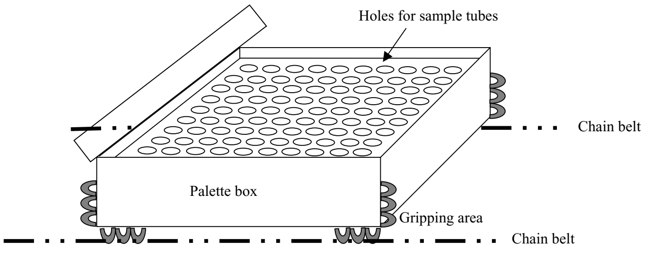

*Figure 6. Sample collection palette.*

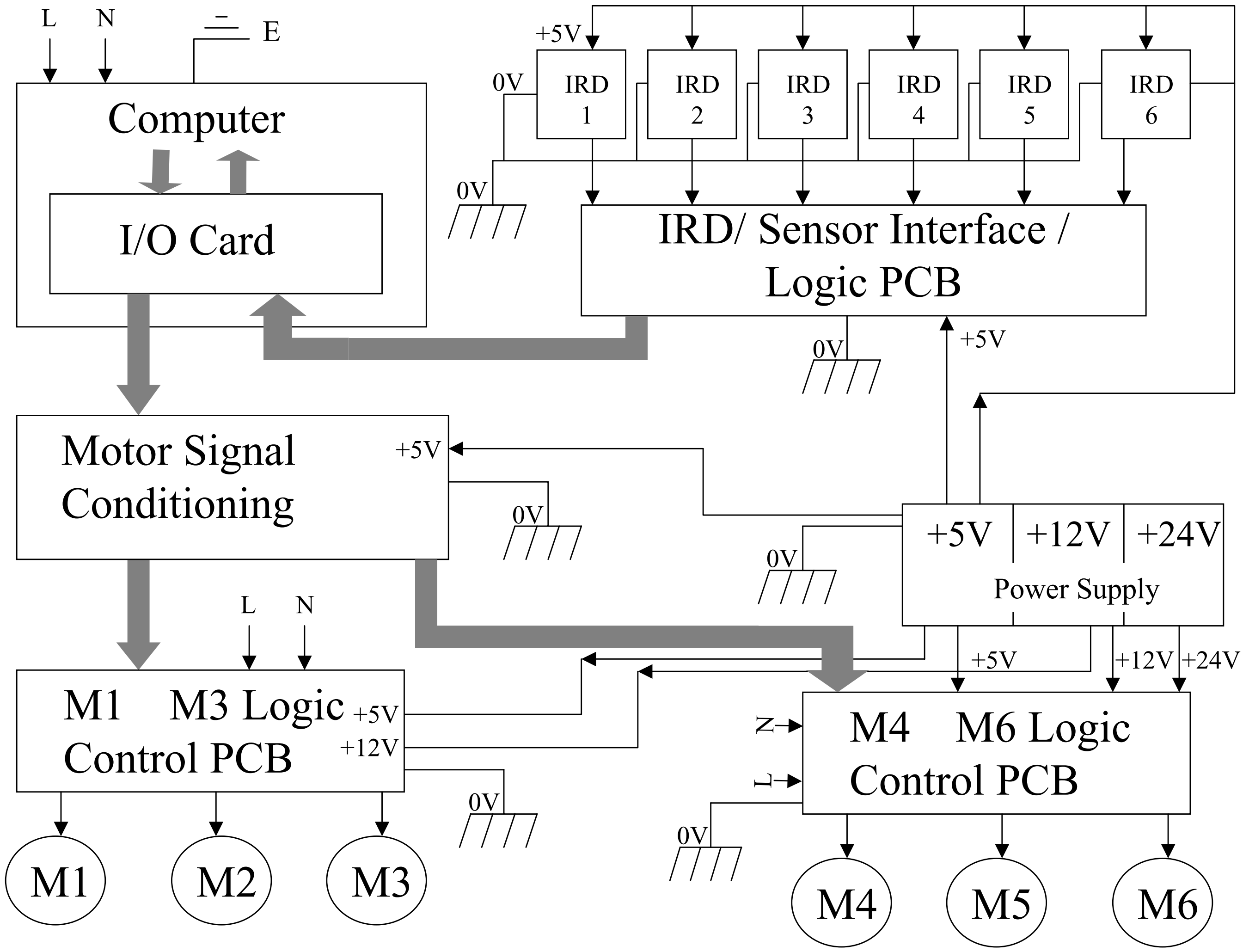

*Figure 7. Schematic of the circuit layout for the automated lysimeter. Motors are labelled M1 to M6, and infrared detectors IRD1 to IRD6.*

Heavy-duty electronic breaks were added to the tower lifting and lowering motors, as the weight of the loaded sample trays would otherwise have caused slippage when the power was released. The main lifting motors were 220 V DC and therefore power was converted from 240 V mains AC using a series of rectifiers. Logic level operations to activate the motors were isolated from the high voltage circuitry to allow computer control of the equipment. Figure 7 is a schematic layout of all the interface circuitry.

The palettes moved from being stored on the left of the apparatus to the sampling area underneath the soil sample, and after a user defined time (usually 4 h) the samples were moved to the right storage tower. Figure 2 shows the movement path of sample palettes around the apparatus. Palettes were tracked using infrared sensors, which were triggered by the palette movement through the infrared detection beams. Infrared sensors were also used to monitor tray position by counting chain links.

Motor activation/deactivation and sensor signals were processed by a DIO24 TTL card (Digital input/output card) feeding signals to a specially written PC based LabVIEW™ software (all National Instruments, Newbury, Berks, UK) virtual console. This console could be run manually or set to automatic operation.

### Results

The results shown in figures 8–10 were of an initial study carried out on the DeBathe soil block. No tracer was added to the soil block, as the data set was used to determine the concentration of phosphate already present in the soil structure. It was also used as a calibration of the sampling regime, i.e. to see if a satisfactory sample collection rate could be achieved and how often sample collection palettes needed to be refreshed. In this study, the sample collection rate was every 4 h.

The results (figures 8–10) showed that there was preferential flow through the soil block, with very little phosphate leaching in the low flow areas. Initial phosphate concentrations were high ($>100\,\mu g\,l^{-1}$ P) before falling rapidly ($<10\,\mu g\,l^{-1}$ P). After 20 h, the phosphate concentration increased again to approximately $50\,\mu g\,l^{-1}$ P.

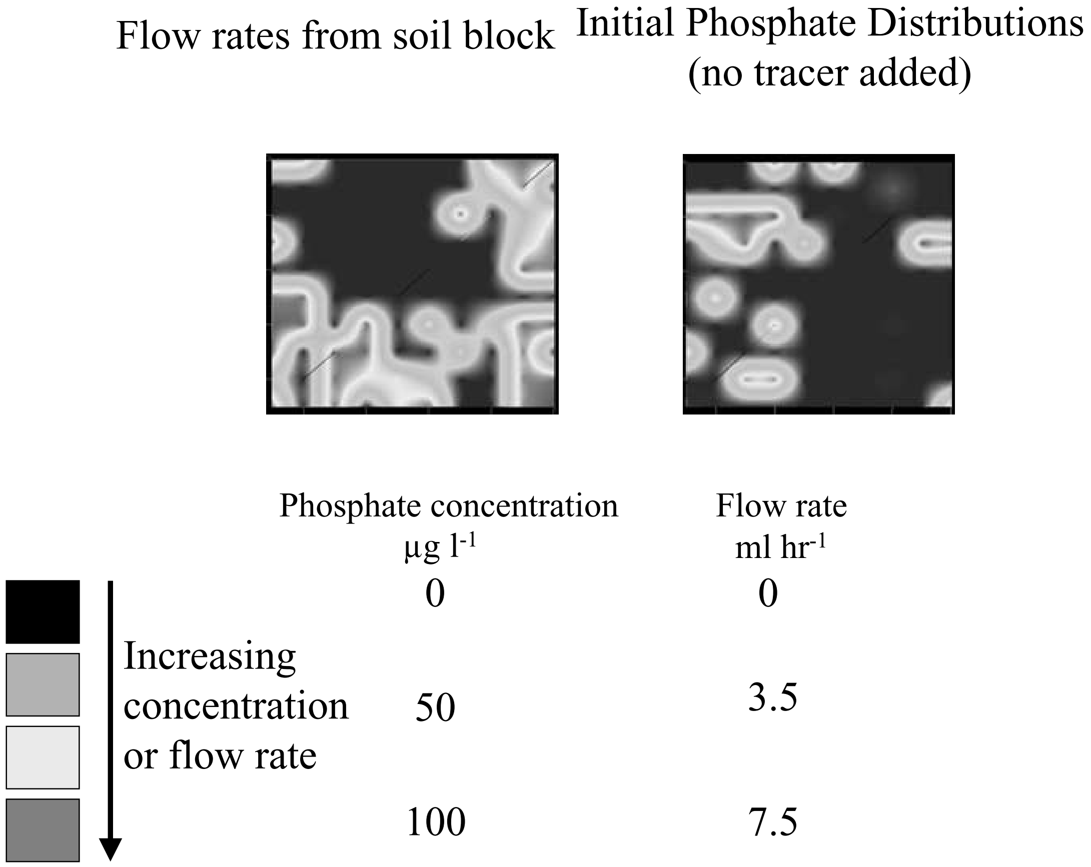

*Figure 8. Experimental results from 0 to 4 h of a DeBathe intact soil block.*

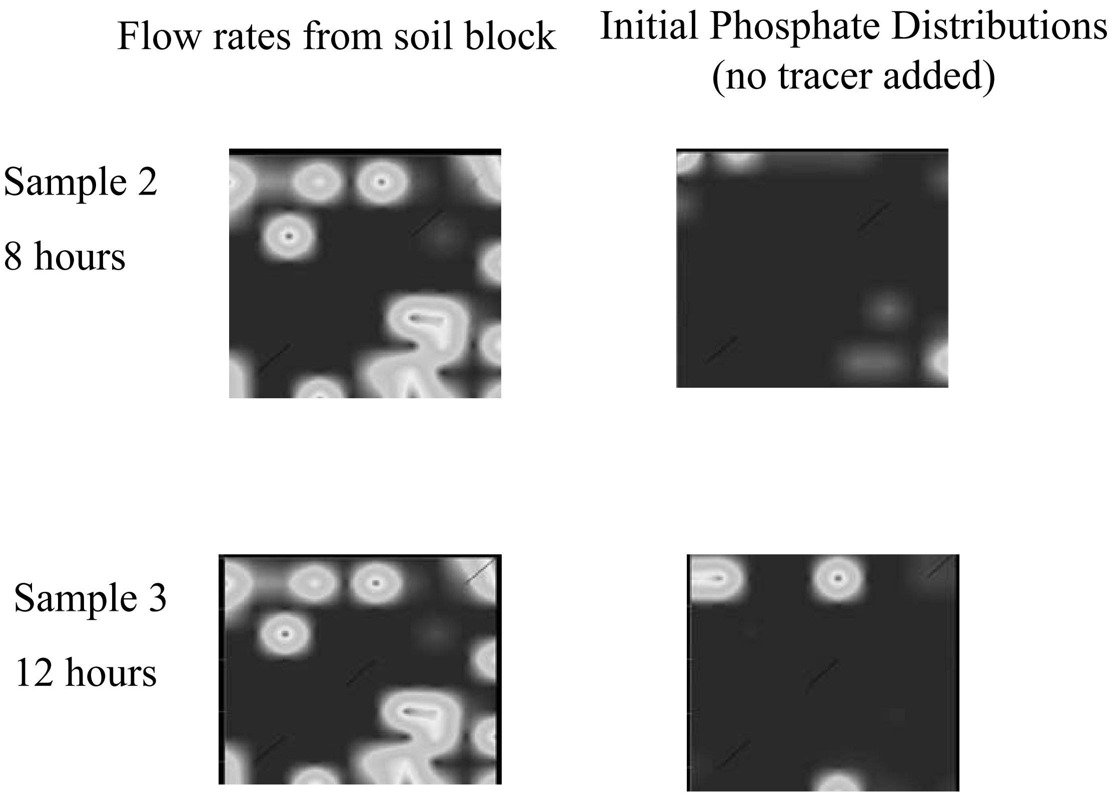

*Figure 9. Experimental results for 4–8 and 8–12 h.*

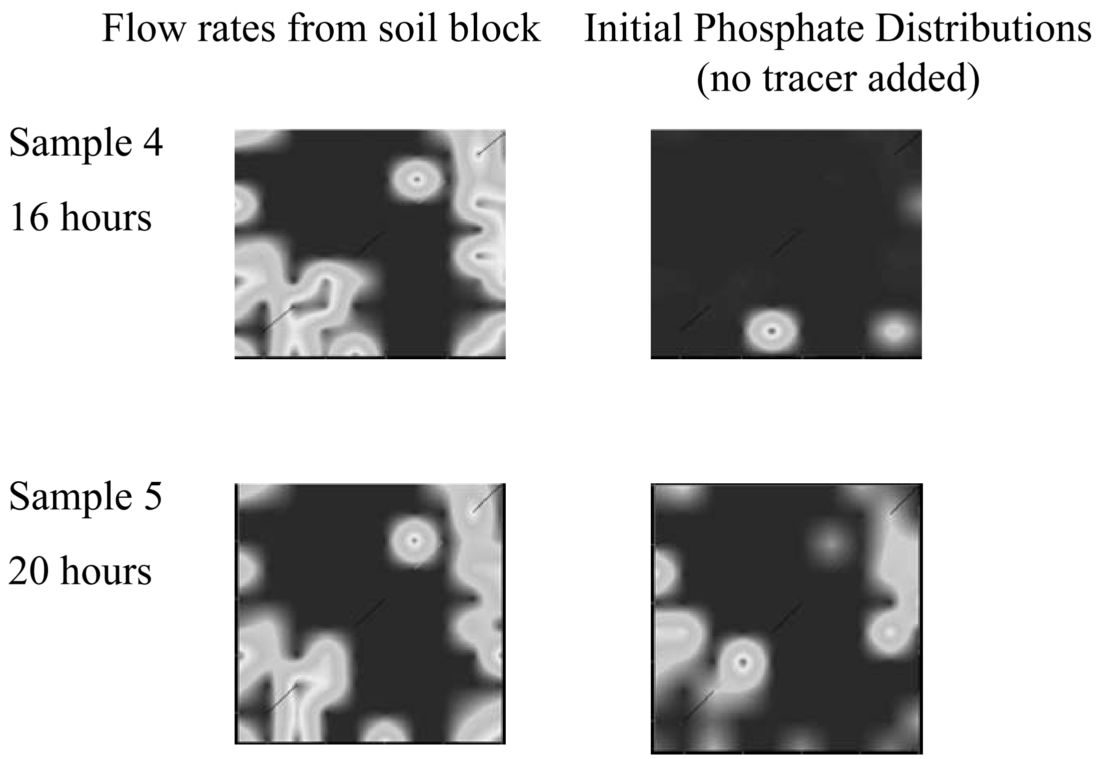

*Figure 10. Experimental results for 12–16 and 16–20 h.*

The initial phosphate plume was due to the presence of dissolved phosphate in the soil matrix water, becoming flushed from the soil block. The second increase was due to the slow removal of sorbed phosphate from soil particulate surfaces. After more than 50 h of flushing and continued monitoring of the eluate the phosphate fell to a concentration less than the detection limit of the air-segmented analyser ($<10\,\mu g\,l^{-1}$ P). This suggested that easily exchangeable phosphate bound by sorption to soil particles had been removed from the block and that any dissolved phosphate was isolated from the preferential flow zones and would not be subsequently leached from the block. A more detailed description of results produced by the application of conservative (bromide) and non-conservative (phosphate) tracers is in progress.

### Conclusions

The combination of automated sample collection and high leachate analysis rates allowed the location of preferential flow zones at the base of the soil block. The phosphate concentration in the leachate was initially high, probably from a mobile water phase, then became very low, and finally after 20 h increased again, this time probably eluting from sorption sites within the matrix. It can also be clearly seen from the diagrams that when phosphate emerged, it only did so in the preferential, high flow-rate channels.

### Acknowledgements

The work was funded by the Engineering and Physical Sciences Research Council and the National Grid Co Plc. The authors are grateful for the design and constructional expertise of Brian Pateman, Adrian Matthews and Ian Rowlands of the University of Plymouth.

### References

1. Schwartz, R. C., McInnes, K. J., Juo, A. S. R. and Cervantes, C. E., *Soil Science*, **164** (1999), 561.
2. Levy, B. S. and Chamber, R. M., *Hydrological Processes*, **1** (1987), 385.
3. Kung, K. J. S., *Geoderma*, **46** (1990), 51.
4. Buchter, B., Hinz, C., Flury, M. and Flühler, H., *Soil Science Society of America Journal*, **59** (1995), 14.
5. Andreini, M. S. and Steenhuis, T. S., *Geoderma*, **46** (1990), 85.
6. Dexter, A. R., *Water Resources Research*, **29** (1995), 1859.
7. Holden N. M., Schofield D., Williams A. G. and Dowd J. F., in *Second Conference on Hydrological Processes in the Catchment*, Krakow, Poland (1995), 239.
8. Zegelin S. J., White I. and Jenkins, D. R., *Water Resources Research*, **25** (1989), 2367.
9. Chow, V. T. and Harbaugh, T. E., *Journal of Geophysical Research*, **24** (1965), 6111.
10. Bowman, B. T., Brunke, R. R., Reynolds, W. D. and Wall, G. J., *Journal Of Environmental Quality*, **23** (1994), 815.
11. Phillips, R. E., Quisenberry, V. L. and Zeleznik, J. M., *Soil Science Society of America Journal*, **59** (1995), 707.
12. Romkens, M. J. M., Glenn, L. F., Nelson, D. W. and Roth, C. B., *Soil Science Society of America Proceedings*, **39** (1975), 158.
13. Mathews, T. J., Void structure, colloid and tracer transport properties of stratified porous media. PhD thesis, University of Plymouth, 1999, 30–41.

